# New Foci of Spotted Fever Group Rickettsiae Including *Rickettsia honei* in Western Australia

**DOI:** 10.3390/tropicalmed1010005

**Published:** 2016-08-17

**Authors:** Edward Raby, Toby Pearn, Andreas G. Marangou, Adam J. Merritt, Ronan J. Murray, John R. Dyer, Stephen R. Graves

**Affiliations:** 1 Fiona Stanley Hospital, Murdoch, WA 6150, Australia; john.dyer@health.wa.gov.au; 2 Recherche Medical Centre, Esperance, WA 6450, Australia; toby_pearn@hotmail.com; 3 Genpar Medical Services, Esperance, WA 6450, Australia; practicemanager.genpar@goldhealth.net.au; 4 PathWest Laboratory Medicine, Nedlands, WA 6009, Australia; adam.merritt@health.wa.gov.au (A.J.M.); ronan.murray@health.wa.gov.au (R.J.M.); 5 Australian Rickettsial Reference Laboratory, Geelong, VIC 3220, Australia; graves.rickettsia@gmail.com

**Keywords:** rickettsia infections/*epidemiology/microbiology, antibodies, bacterial/*blood, tick infestations/*complications, Western Australia/epidemiology

## Abstract

We describe the first reported case of spotted fever group rickettsiosis in Western Australia, and two cases of probable *Rickettsia honei* from a new geographic focus. These findings highlight the need to raise awareness of ricksettsial infection among local clinicians as well as those treating visitors to this region, important for outdoor recreation.

## 1. Introduction

Western Australia (WA) occupies the western third of Australia, covering an area of over two and a half million km^2^ ranging from the tropical north to the temperate south and with a large eastern desert region. Flea-borne endemic (murine) typhus due to *Rickettsia typhi* has been recognised in WA since the early 20th century with up to 140 cases a year notified in the period after the First World War [1], with isolated cases and small clusters reported more recently [2]. A focus of scrub typhus due to mite-borne *Orientia tsutsugamushi* is found in the tropical north of the state [3]. Serosurveys have suggested exposure to spotted fever group (SFG) rickettsia in the northern Kimberley region and more recently in those engaging in adventurous outdoor pursuits around the metropolitan area [3,4]. Rickettsial DNA has been detected in ectoparasites (ticks and fleas) removed from humans and animals including *Rickettsia felis,* and the novel SFG rickettsia with the proposed name “Rickettsia gravesii” was found in WA bush fauna throughout the southwest of the state as well as on Barrow Island in the central coastal region [5,6,7]. However, prior to this report, no human cases of SFG rickettsiosis have been confirmed in WA.

## 2. Clinical Records

### 2.1. Case A

**Figure 1:**
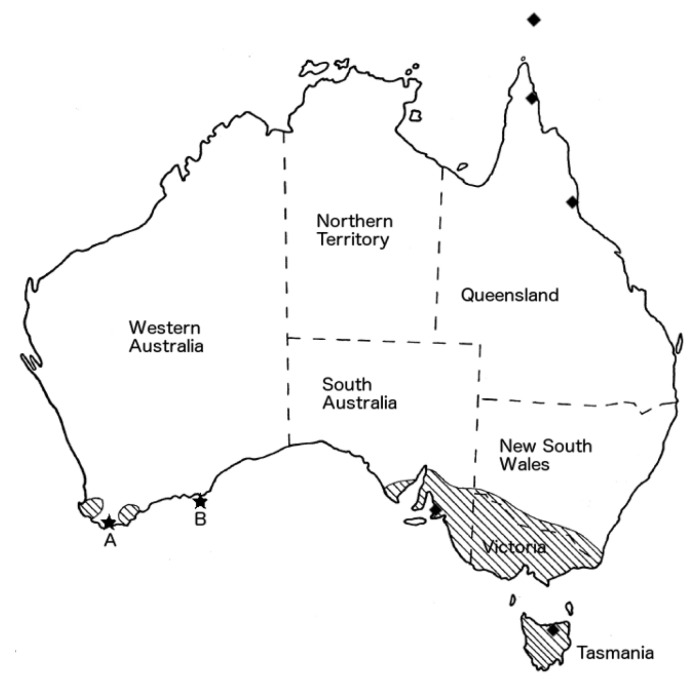
Map of Australia with stars showing location of acquisition of rickettsial infection for cases A (Walpole) and B (Salisbury Island). Shaded regions show the known range of *Bothriocroton hydrosauri* which in South Australia and Victoria overlap with the identified range of *Rickettsia honei*. Diamonds localise the reported cases of *R. honei* marmionii strain in Australia and the Torres Strait Islands [8]. Adapted with permission from [9] (Figure 37).

A 51-year-old man presented in mid-May (early winter) 2006, eight days after onset of swollen groin lymph nodes followed three days later by fevers, rigors, headache, dry cough and subsequent non-pruritic rash. He had been hiking alone along a popular hiking trail, the Bibbulmun track, in the southwest of WA and had received numerous tick bites. A tick removed from the skin of his back after biting near Walpole (Figure 1) was identified as *Ixodes australiensis*, a tick indigenous to WA and Tasmania.

**Figure 2:**
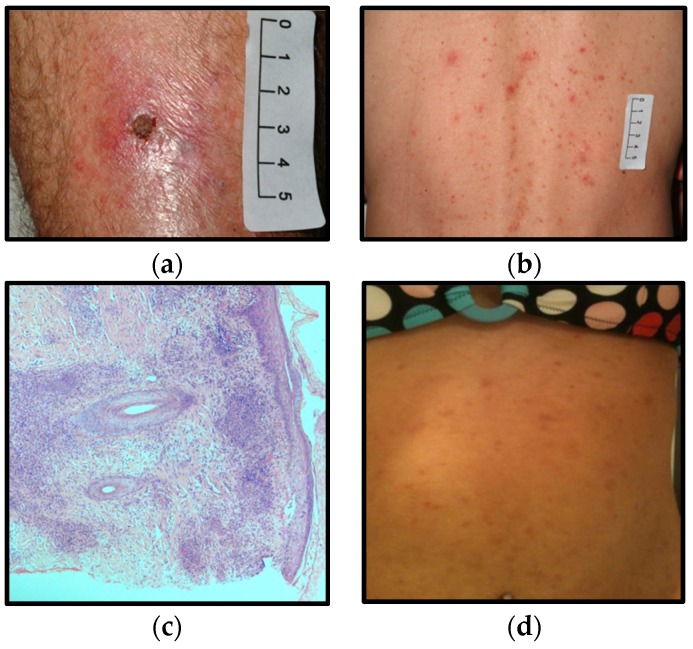
Clinical signs and histopathological findings from cases A (**a**–**c**) and B (**d**): (**a**) lower limb eschar; (**b**) papulovesicular rash on trunk; (**c**) hematoxylin and eosin stained biopsy of eschar; (**d**) maculopapular rash on abdomen.

On examination, he did not appear acutely unwell but was febrile. There were multiple tick bites on the trunk and limbs, a sparsely distributed papulovesicular rash on the trunk and extremities, an eschar overlying the left tibia (Figure 2) and pronounced inguinal lymphadenitis. Full blood count was normal but for moderate lymphopenia; there was moderate elevation of alanine transaminase (ALT) to 177 U/L, increasing to 430 U/L one week later, and C-reactive protein (CRP) was 40 mg/L. Chest X-ray was normal. He was prescribed doxycycline 100 mg bd for seven days. Within 24 h there was significant symptomatic improvement and two weeks later he was completely well with healing of the eschar and normalisation of CRP.

Citrate synthase gene PCR specific for rickettsiae [10] was performed at the Australian Rickettsial Reference Laboratory (ARRL) on a biopsy taken from the edge of the eschar the day after the commencement of the doxycycline. This confirmed the presence of the *Rickettsia* genus, but there was insufficient product for DNA sequencing and species identification. Histopathological examination revealed florid perivascular granulomatous inflammation (Figure 2). PCR on the whole blood was negative. Serological testing in this and subsequent cases was performed by immunofluorescence assay with commercial slide preparations (Scimedx Corp, Dover, NJ, USA) using *R. conorii* (Moroccan), *R. typhi*, and *O. tsutsugamushi* (Karp strain) to represent the spotted fever group, typhus group, and scrub typhus group respectively, using a polyvalent conjugate to detect total antibody (IgG and IgM). Serum tested in parallel showed evidence of acute infection with SFG rickettsia (Table 1). Rickettsial organisms were unable to be isolated from the tick retrieved from the patient’s skin and PCR for rickettsial DNA was also negative.

### 2.2. Case B

A 36-year-old female spent four days during November (early summer) 2014 on a field trip to Salisbury Island, situated in the Recherche Archipelago approximately 150 km east of Esperance (Figure 1), trapping black-flanked rock wallabies and carrying out botanical inventory work. On return to the mainland she discovered a tick, which she promptly removed. Three days after her return she complained of fatigue and headache, followed three days later by fevers with rigors and general malaise. A maculo-papular rash (Figure 2) over the limbs, trunk and face started to emerge the following evening accompanied by myalgia and arthralgia. Four days into her febrile illness she was prescribed doxycycline 100 mg bd. Her fevers and arthritis abated within 48 h. Malaise, fatigue and myalgias took more than three weeks to resolve. During the acute phase she developed mild thrombocytopenia (104 × 10^9^ platelets/L). The white cell count remained normal while the CRP rose to 256 mg/L and she had a mild hepatitis (ALT 87 U/L). Seroconversion to SFG rickettsia was demonstrated (Table 1).

DNA was extracted from her acute phase serum and the rickettsial 17 kD antigen gene was amplified and sequenced using primers MTO1 and MTO2 [11]. A 429 base pair (bp) sequence was generated (GenBank sequence ID: KU521358.1) which showed 100% match to the *R. honei* strain RB (411/411 bp; AF060704.1) with a 4 bp difference from the *R. honei* marmionii strain (421/429 bp; KT032120.1) and a single bp mismatch with “R. gravesii” (393/394 bp; DQ269436.1). Attempts to amplify and sequence the 16S rRNA gene from the limited volume of residual serum were unsuccessful.

### 2.3. Case C

Another member of Case B’s field trip party was identified in convalescence, having suffered similar symptoms shortly after the return from Salisbury Island. When Case C was first seen four weeks after onset of symptoms, his fevers had resolved but he complained of residual fatigue and myalgia. At this stage the CRP was normal but he had mild residual hepatitis (ALT 103 U/L). He received doxycycline with symptomatic improvement. Rickettsial DNA was not detectable in the whole blood. No tick was recovered. Serial serology at four and 12 weeks (Table 1) showed a rising titre to SFG. Intriguingly, he was also diagnosed at that time with Graves’ disease, which has been discussed in relation to this case elsewhere [12].

## 3. Discussion

Spotted fever group rickettsiosis is widespread on the eastern seaboard of Australia, manifesting as either Queensland tick typhus or Flinders Island spotted fever. Queensland tick typhus due to *R. australis* is found from the tip of southeastern Australia through to northern Queensland and the Torres Straits islands [13]. *Rickettsia australis* is transmitted by *Ixodes holocylcus* and *I. tasmani* from vertebrate hosts including native rats and bandicoots. Flinders Island spotted fever has a more patchy distribution, with the classical *R. honei* strain RB being found on Flinders Island to the northeast of Tasmania [14,15]. In Australia, *R. honei* is transmitted by *Bothriocroton hydrosauri* from native reptiles including blue-tongue lizards and snakes. The closely related *R. honei* strain marmonii has been identified in areas of coastal Queensland and the islands of the Torres Straits [8]. *Rickettsia honei* cases have also been described in the coastal region southeast of Adelaide as well as in isolated cases in Thailand and Nepal [8,16,17]. *Rickettsia australis* and *R. honei* have yet to be discovered in the tick population of WA.

“Rickettsia gravesii” appears to be widespread in WA and has been found in tick species that live on macropods but are known to bite humans. “Rickettsia gravesii” is not yet fully characterised but has been maintained in culture, allowing the analysis of its genome, which has shown that it is closely related to the pathogenic SFG *Rickettsia massiliae* [18]. Rickettsial DNA with a citrate synthase gene homologous to “R. gravesii” has been detected in *Amblyomma postoculatum* from Barrow Island [19]. DNA consistent with “R. gravesii” was highly prevalent in the voracious human feeder *A. triguttatum* removed from animals and humans on Barrow Island as well as from feral pigs and other opportunistic samples from throughout the southwest of the state [5,6,20]. This suggests that the most likely infective agent in Case A was “R. gravesii”, although, given transmission occurred in a geographically isolated area and the tick removed was *I. australiensis*, the infection may well have been caused by a novel species.

Limited genotypic analysis of rickettsial DNA from Case B most closely matched *R. honei*, and given the epidemiological link, Case C is also likely to be due to this agent. Whereas other SFG rickettsiosis worldwide, including *R. australis* in Australia and *R. honei* in Asia and America, have been associated with mammalian ectoparasites, the southern reptile tick *Bothriocroton hydrosauri* has been identified as the likely vector of *R. honei* in Australia [21]. Case B believed the tick she removed was *A. triguttatum*, the common kangaroo tick, but unfortunately it was discarded and not formally identified and the invertebrate fauna of this region has not yet been well characterised.

The finding of rickettsiae on this remote island expands the south-western range of the organism and suggests both its historical and likely current presence on the adjacent mainland, which is sparsely inhabited but an important region for outdoor recreation. Further surveys are required to verify the presence of *R. honei* and define the ecology and vectors in this region as well as to confirm the infectious threat of “R. gravesii”. In the interim, it is important to raise awareness of rickettsiosis among local clinicians as well as those who treat visitors to the area.

## Figures and Tables

**Table 1:** Rickettsial antibody titres by immunofluorescence assay according to time after onset of illness.

		Day 7/8	1 Month	2 Months	3 Months	2 Years	7 Years
Case A	SFG	<128	128	16,384		128	<128
	TG	<128	<128	256		<128	<128
	STG	<128	<128	<128		<128	<128
Case B	SFG	<128	2048				
	TG	<128	<128				
	STG	<128	<128				
Case C	SFG		128		512		
	TG		128		128		
	STG		<128		<128		

SFG: spotted fever group; TG: typhus group; STG: scrub typhus group.
